# Gallbladder non-Hodgkin’s lymphoma: Case report

**DOI:** 10.1016/j.ijscr.2019.07.064

**Published:** 2019-07-25

**Authors:** Fei Gao, Haichao Zhao, Xidong Chen, Xiushan Dong, Tao Liu, Xifeng Fu

**Affiliations:** a Shanxi Academy of Medical Sciences Shanxi Dayi Hospital, Longcheng Street 99, Taiyuan, Shanxi, 030032, China; b Graduate School of Shanxi Medical University, Xinjian South Road 56, Taiyuan, Shanxi, 030032, China

**Keywords:** Extrahepatic biliary non-Hodgkin’s lymphoma, Hematopoietic syndrome, Bile duct disappearance syndrome, Case report

## Abstract

**Introduction:**

Extrahepatic biliary non-Hodgkin’s lymphoma (EBNHL) is rare, with a prevalence of 0.6% of malignant biliary tumors. Primary biliary non-Hodgkin’s lymphoma accounts for 0.4% of extranodal non-Hodgkin’s lymphoma (NHL), accounting for approximately 0.016% of all NHL cases. The patient presented with typical bile duct disappearance syndrome. The late performance is a bloodthirsty syndrome.

**Presentation of case:**

This patient was admitted to the hospital with “intermittent fever, abdominal pain for 19 days”. The relevant laboratory tests showed severe infection. The patients were treated according to abdominal infection, and then laparoscopic cholecystectomy was performed. After the operation, the patient's condition did not relieve and further deteriorated. Postoperative patient performance was not based on preoperative estimates, and surgery did not achieve the desired results. Postoperative pathological diagnosis confirmed gallbladder non-Hodgkin’s lymphoma. The patient eventually died.

**Discussion:**

Non-calculus inflammation occurs more frequently in the gallbladder. Non-calculus infections are generally caused by bile stasis and ischemia, resulting in bacterial reproduction and reduced blood supply, and gangrene and perforation of the gallbladder are more likely to occur. This patient is also diagnosed and treated according to this idea. The development after surgery completely exceeded our expectations. Gallbladder non-Hodgkin’s lymphoma is very rare, and this case also includes hemophagocytic syndrome and bile duct disappearance syndrome, which is rare.

**Conclusion:**

Malignant lymphoma of the extrahepatic biliary system is extremely rare, and its clinical manifestations are easily misdiagnosed. At the same time, non-calculous cholecystitis with fever, jaundice and hepatosplenomegaly should be considered.

## Highlights

• The patient was hospitalized for “intermittent fever, abdominal pain for 19 days” and then underwent laparoscopic cholecystectomy. After the operation, the patient’s condition deteriorated and eventually died. Postoperative pathological diagnosis confirmed gallbladder non-Hodgkin’s lymphoma.
• Gallbladder non-Hodgkin’s lymphoma is very rare, and this case also includes hemophagocytic syndrome and bile duct disappearance syndrome, which is rare. This case is described from the onset of the patient to the hospital visit and postoperative conditions.
• Bile duct disappearance syndrome (VBDS) is a syndrome characterized by reduced intrahepatic bile ducts as a pathological feature and cholestasis as the main clinical manifestation.
• Hemophagocytic syndrome (HPS) is a group of heterogeneous diseases characterized by over-inflammatory responses due to hereditary or acquired immunodeficiency.

## 1 Introduction

Extrahepatic biliary non-Hodgkin’s lymphoma (EBNHL) is rare. In 2007, Odemis et al. [1] retrospective study reported that 7 of the 1123 patients with malignant cholangiocarcinoma were diagnosed with biliary non-Hodgkin’s lymph Tumors (0.6%), and primary biliary non-Hodgkin’s lymphoma accounted for 0.4% of extranodal NHL, accounting for only about 0.016% of all NHL cases. Due to the infiltration of malignant lymphoma cells, the biliary system has a wide range of pseudo-inflammatory manifestations (including gallbladder and bile ducts). It is expressed as fever and jaundice. The imaging manifestation is a thin line change of the bile duct, which is called bile duct disappearance syndrome. At the same time, non-Hodgkin’s lymphoma causes hyperproliferation of T cells and macrophages, which are manifested as persistent fever, hepatosplenomegaly, and complete blood cell reduction, called bloodthirsty syndrome. Our work has been reported in line with the SCARE criteria [2].

## 2 Presentation of case

Male patient, adult, Chinese, Han nationality. The patient had chills and high fever at home, and the body temperature was up to 40 °C. At the same time, there was a dull pain, which was in the middle and upper abdomen. He was admitted to the local hospital respiratory department and was diagnosed as “pulmonary infection, emphysema, and bullae.” The doctor gave him anti-inflammatory, symptomatic treatment. His abdominal pain gradually improved, but the fever did not improve. At that time, the cockroach test was cultured as Candida albicans, and it was suspected that there was a “pulmonary fungal infection.” The doctor gave him antifungal treatment, but the treatment was poor. Later, he was admitted to the respiratory department of our hospital and was admitted to hospital with “fever cause for investigation”.

**Fig. 1:**
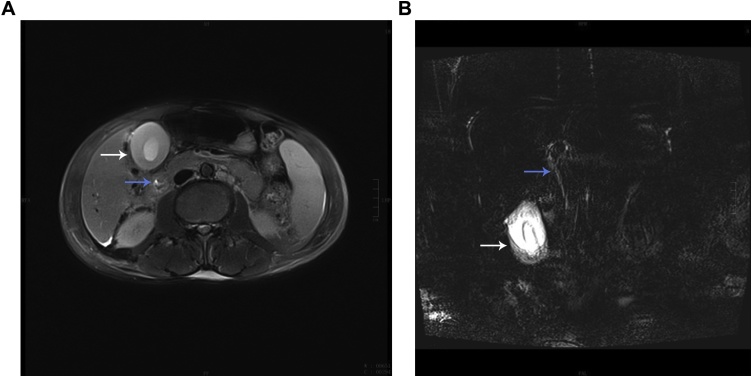
MRCP **A** The gallbladder wall was significantly thickened to a thickness of 1.47 cm. There was no acute inflammatory change in the gallbladder (white arrow); no bile duct dilatation, no stones in the gallbladder, thickening of the bile duct wall, and inflammation (blue arrow); **B** The gallbladder cavity is reduced (white arrow), the bile duct is 6.0 mm wide, and the lumen is changed in a thin line (blue arrow).

Admission test showed: blood cell analysis: WBC 2.7 × 10^9^/L, RBC 4.56 × 10^12^ / L, PLT 38 × 10^9^/L; tumor markers: CA125 1269.9U/mL, ferritin >1500 ng/mL, CA50 34.5IU/mL, CA199 107.4 U/mL; liver function: STB 181.8 μmol/L, CB 116.5 μmol/L, UCB 65.3 μmol/L, ALT 150 IU/L, AST 156.6 IU/L. Coagulation examination: PT 16.4 s, APTT 41.9 s, D-dimer 1787 ng/mL. CT of the chest and abdomen: infertility of the upper lobe of the right lung; emphysema, pulmonary alveolar; edema of the gallbladder wall, edema of the portal vein; no obvious dilatation of the intrahepatic bile duct; pelvic fluid. MRCP (Fig. 1): acute cholecystitis, thickening of the gallbladder wall (1.47 cm), no stones in the gallbladder cavity; no obstruction in the biliary tract; full liver morphology, increased spleen volume; effusion in the perihepatic and spleen weeks. There were no obvious abnormalities in the routine examination before surgery. Preoperative diagnosis: the nature of the right lung lesions to be investigated Pneumonia? Lung cancer? Chronic obstructive pulmonary disease Chronic pulmonary heart disease; Old tuberculosis; Causes of jaundice to be investigated Acute acalculous cholecystitis Acute obstructive cholangitis? Impaired liver function.

On the second day of admission, due to the serious uncontrollable symptoms of the patient's infection, cholecystectomy was performed under general anesthesia. During the operation, a small amount of pale yellow effusion was seen in the abdominal cavity, the thickness of the gallbladder was thick and edematous, the diameter of the common bile duct was 6–8 mm, the bile duct was not paralyzed, and the stones and tumor were given. The intraoperative platelet was given 1 U, and the operation time was about 60 min. Return to the ward after surgery.

**Fig. 2:**
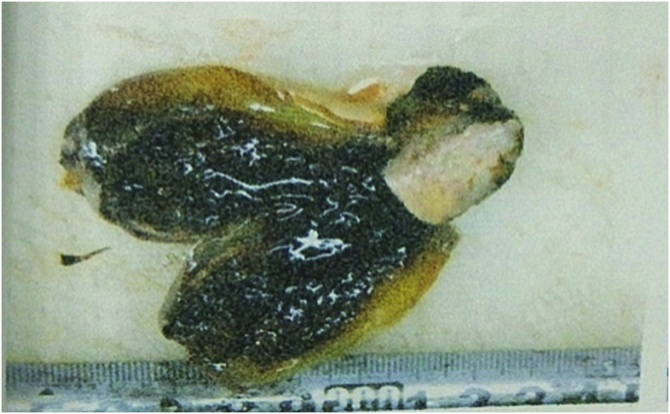
Gallbladder solid specimen (visual view) Generally seen: size about 8.0 × 3.0 cm, gallbladder wall is pale, wall thickness edema, gallbladder cavity is significantly reduced, a small amount of bile can be seen in the cavity.

**Fig. 3:**
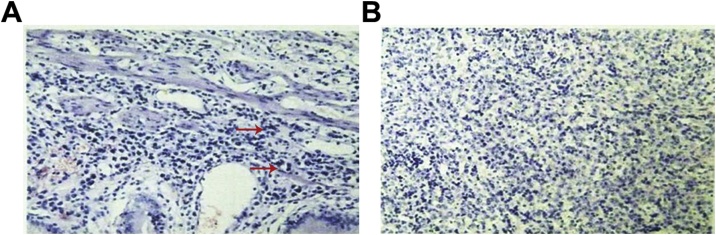
Pathological manifestations of gallbladder surgery specimens (HE staining, ×200). Microscopically seen: HE staining showed atypical lymphocytes (red arrow); lamina propria of the gallbladder mucosa and muscle layer scattered in atypical lymphoid cells, see mitotic figures.

**Fig. 4:**
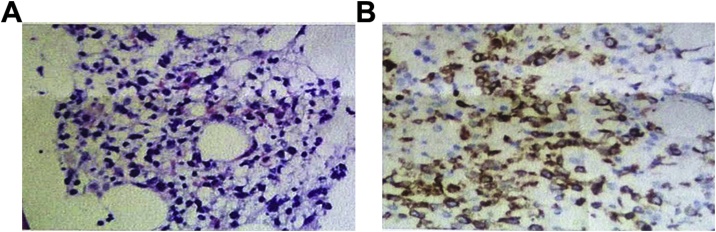
Bone marrow biopsy results. **A** (HE staining, 10 × 40): A small amount of bone marrow hematopoietic tissue can be seen, lymphoid cells are scattered and karyotype is irregular; **B** (immunotactic CD3, 10 × 40): labeling result CD3(+), CD235a erythroid (+), MPO granule (+), CD2 (+), CD56 (+), EBER (+).

Postoperative patients still had intermittent fever, body temperature up to 39.1 °C, breathing 20–28 times / min, pulse 108–120 beats / min, blood pressure 120–130/70–90 mmHg; laboratory tests showed that white blood cells, platelets continued to reduce, gallbladder The erythropoietin and transaminase continued to increase, the clotting test PT prolonged, and the D-dimer increased. Abdominal ultrasound at the bedside: The extrahepatic bile duct was not developed, and the intrahepatic bile duct showed a thin line appearance. No stones were found, no effusion in the gallbladder fossa. Bone marrow puncture smear can see atypical lymphocytes. Pathology: gross observation: the gallbladder size is about 8.0 × 3.0 cm, the gallbladder wall is pale, the wall thickness is edema, the gallbladder cavity is obviously reduced, and a small amount of bile is visible in the cavity. Pathological results (Fig. 2, Fig. 3) showed that atypical lymphocytes were seen by HE staining of gallbladder; lamina propria and muscle layer of gallbladder mucosa were infiltrated with atypical lymphoid cells, see mitotic figures; immunohistochemical markers: interstitial cells: Ki67 > 80%, CD3 (+); pathological diagnosis: malignant tumor of the gallbladder lymphoid hematopoietic system. Bone marrow biopsy results (Fig. 4) suggest a diagnosis of bone marrow non-Hodgkin’s lymphoma, NK/T cell tumor. The EB virus nucleic acid assay was 1.88 × 105 copy/mL. Final diagnosis: biliary non-Hodgkin’s lymphoma EB virus infection Hemophagocytic syndrome Liver failure. Five days after surgery, the patient died.

## 3 Discussion

Early manifestations of the patient suggest an infection. Post-hospital examinations suggest extrahepatic biliary system infections, mainly considering gallbladder and biliary infections. Because the patient's infection is severely uncontrollable, acute acalculous cholecystitis is easy to perforate in the early stage due to early surgical treatment. At the same time, however, the physician considered the acute inflammation of the gallbladder to cause bile duct inflammation, and early neglected the imaging findings of bile duct thinning. Postoperative pathology confirmed a malignant lymphatic system tumor of the gallbladder. Bone marrow smears indicate that the patient is a non-Hodgkin’s lymphoma. Therefore, the patient is considered to be a non-Hodgkin’s lymphoma of the gallbladder. Because only the specimen of the gallbladder was obtained by surgery, no bile duct tissue was obtained, but based on the patient's performance and examination suggestion, we inferred that the patient had non-Hodgkin’s lymphoma of the extrahepatic biliary tract.

Extrahepatic biliary non-Hodgkin’s lymphoma (EBNHL) is rare. In a 2007 Odemis et al. retrospective study [1], 7 of 1123 patients with malignant cholangiocarcinoma were diagnosed with biliary non-Hodgkin’s lymph Tumors (0.6%), primary biliary non-Hodgkin’s lymphoma accounted for 0.4% of extranodal NHL, accounting for only about 0.016% of all NHL cases. The clinical manifestations are jaundice, upper abdominal pain, fever, weight loss, etc.; jaundice is common, and most of them are caused by obstruction of extrahepatic bile ducts, some appear as first symptoms; biliary colic as a clinical symptom is rare. Liver function: biliary obstruction, total bilirubin, elevated transaminase. Blood cell analysis: white blood cell count, platelet count decreased. Coagulation test: prolonged clotting time. Imaging findings (such as MRCP, CT) and cholangiocarcinoma performance are very similar, common extrahepatic bile duct wall thickening, stenosis, proximal bile duct dilatation; intrahepatic bile duct showed fine line appearance. This patient with fever, abdominal pain, jaundice as the main clinical manifestations, may be due to bile duct obstruction leading to acute cholangitis, but fever also need to consider other related issues of the blood system; MRCP showed that the intrahepatic bile duct showed fine line appearance, the gallbladder cavity volume was significantly reduced small.

The bile duct disappearance syndrome (VBDS) is a syndrome in which intrahepatic bile duct reduction is a pathological feature and cholestasis is the main clinical manifestation. Common causes are developmental metabolism, lymphoma, vascular disease, immunity, and drugs. The main clinical manifestations were abdominal discomfort, jaundice, itchy skin, steatorrhea, elevated transaminase, bilirubin and alkaline phosphatase; MRCP and ERCP showed signs of disappearance of intrahepatic bile duct, no bile duct tree branches in the liver, and left and right hepatic ducts showed only a few branches; the diagnosis needs to rely on liver biopsy [3,4]. In the literature, intraoperative bile duct wall thickening, narrow lumen, difficult to retain T tube. In this case, the gallbladder wall was abnormally thickened, and the intraoperative common bile duct was not widened. The test showed jaundice, but postoperative MRCP and ultrasound indicated that the intrahepatic and extrahepatic bile ducts changed or disappeared, which may be caused by diffuse infiltration of the biliary system.

Hemophagocytic syndrome (HPS), also known as Hemophagocytic lymphohistiocytosis, was first reported by Risdall et al. in 1979 [5]. It is a group caused by hereditary or acquired immunodeficiency. A heterogeneous disease characterized by an excessive inflammatory response. The clinical features of persistent fever, hepatosplenomegaly, whole blood cell reduction, and bloodthirsty in bone marrow, liver, spleen, and lymph node tissues are the main features. Secondary HPS is more common in EB virus infection and non-Hodgkin’s lymphoma. The main treatments were high-dose dexamethasone, etoposide and cyclosporine-A; the disease progressed rapidly and the prognosis was poor [6]. The patient presented with severe infection at admission, with decreased white blood cells, red blood cells, and platelets, and further decline after surgery, considering HPS. However, due to lack of timely judgment, no relevant treatment was given.

We should reflect on this patient. Early consideration of extrahepatic biliary tract inflammation in patients, acute acalculous cholecystitis and cholangitis were considered according to imaging examination. No symptoms were improved after early surgical treatment. We invited respiratory, infectious, and intensive medicine departments to conduct consultations and consulted in large numbers. In the literature, blood system malignancies were considered after the blood department was invited to consult because of the progressive decline of the three lines of cells. The patient has died before the pathological examination and the bone marrow smear results are clearly diagnosed. There is no timely treatment for hematological malignancies.

The clinical manifestations and auxiliary examinations of this case were consistent with the diagnosis of EBNHL, VBDS and HPS. Such cases are often misdiagnosed as acute acalculous cholecystitis, acute suppurative cholangitis, and cholangiocarcinoma due to their special performance. The misdiagnosis rate is about 93% [7,8]. Therefore, the diagnosis of primary extrahepatic bile duct NHL is rare in the operation. Therefore, the surgical methods usually include bile duct exploration and drainage, cholangiocarcinoma radical resection, and pancreaticoduodenectomy. Due to the high rate of misdiagnosis before surgery, the vast majority of patients have undergone surgery before the diagnosis.

## 4 Conclusion

Malignant lymphoma of the extrahepatic biliary system is extremely rare, and its clinical manifestations are easily misdiagnosed. At the same time, non-calculous cholecystitis with fever, jaundice and hepatosplenomegaly should be considered.

## Sources of funding

Funding in the department.

## Ethical approval

The authors declare that all procedures followed were in accordance with the ethical standards of the responsible committee on human experimentation (institutional and national). Informed consent was obtained from the patient for being included in the study.

## Consent

Written informed consent was obtained from the patient’s family for publication of this case report and accompanying images. A copy of the written consent is available for review by the Editor-in-Chief of this journal on request.

## Author contribution

Doctor Gao Fei is the competent doctor of the patient in this case report. Zhao Haichao writes the article. Doctor Liu Tao conducts data sorting. Director Dong Xiushan and Director Fu Xifeng is responsible for guiding the writing of the article.

## Registration of research studies

This article does not need Unique Identifying Number (UIN).

## Guarantor

All authors of the article are responsible for this article and are responsible for this.

## Provenance and peer review

Not commissioned, externally peer-reviewed.

## Declaration of Competing Interest

No conflict of interest.
